# Prospective evaluation of uterine artery embolization using diluted NBCA-lipiodol mixture for uterine fibroids: preliminary treatment outcomes

**DOI:** 10.1186/s42155-026-00782-9

**Published:** 2026-10-07

**Authors:** Nguyen Thi Bich Van, Nguyen Thanh Khiem, Nguyen Ngoc Cuong, Phan Hoang Giang, Nguyen Quang Trung, Thieu Thi Tra My, Bui Thi Phuong Thao, Tran Quoc Hoa

**Affiliations:** 1 Department of Obstetrics, Hanoi Medical University Hospital, Hanoi, Vietnam; 2 Department of Obstetrics, Hanoi Medical University, Hanoi, Vietnam; 3 Radiology Center, Hanoi Medical University Hospital, Hanoi, Vietnam; 4 Department of Radiology, Hanoi Medical University, Hanoi, Vietnam; 5 Department of Thyroid, Vinmec Times City International Hospital, Vinmec Healthcare System, Hanoi, Vietnam; 6 Urology department, Hanoi Medical University, Hanoi, Vietnam

**Keywords:** Uterine artery embolization, Uterine fibroids, NBCA, Liquid embolic agent, Transradial access

## Abstract

**Background:**

This study aimed to determine the safety and effectiveness of uterine artery embolization using NBCA – lipiodol mixture via radial artery access for the treatment of uterine fibroids.

**Methods:**

Forty patients underwent uterine artery embolization with NBCA mixed with lipiodol (1:10). The uterine fibroid volumes were determined on magnetic resonance imaging before and 3–6 months after the procedure. We analyzed the following parameters: dominant fibroid volume, hemoglobin levels, symptoms before treatment, after embolization, and complications.

**Results:**

The mean age was 41 ± 6.5 years and mean uterine volume prior to treatment, 137.7 ± 104.3 ml. After 3–6 months embolization, the mean fibroid volume decreased from 137.7 ± 104.3 cm^3^ to 51 ± 54.3 cm^3^ (p < 0.001), corresponding to a mean volume reduction of 64.7 ± 22.7%. Hemoglobin levels also significantly increased (p < 0.001). No major intraprocedural adverse events were observed. Major complications occurred in 2.5% (fibroid expulsion). The minor complications rate was 20%, related to post-embolization syndrome (pain). The late adverse events occurred in 10%: alopecia (7.5%), vaginal dryness (2.5%). The length of hospital stay was 1.9 ± 0.8 days. Clinical symptomatic improvement was observed in 100% and symptoms were resolved in 87.5%.

**Conclusion:**

This prospective single-arm study suggests that UAE using a diluted NBCA–lipiodol mixture is feasible and appears to be safe, with encouraging early clinical and MRI outcomes. However, the short follow-up period, absence of a comparative control group, and lack of direct assessment of fibroid infarction preclude definitive conclusions regarding its efficacy or its role relative to established particulate embolic agents.

## Introduction

Uterine fibroids are a highly prevalent benign condition with heterogeneous clinical presentations [1]. Contemporary management strategies emphasize symptom control, uterine preservation, and minimization of treatment-related morbidity [1, 2]. Rather than a single optimal therapy, individualized treatment selection—often favoring minimally invasive approaches such as uterine artery embolization—represents the current standard of care. Clinical outcomes after uterine artery embolization (UAE) are strongly influenced by the choice of embolic material, which determines the extent and durability of arterial occlusion [3]. The evolution of embolic materials used in uterine artery embolization has progressed from temporary agents such as gelatin sponge to permanent particulate embolics, including polyvinyl alcohol particles and calibrated microspheres, which remain the most widely used in clinical practice [1, 3, 4]. Advances in embolization strategies have therefore led to growing interest in liquid embolic agents capable of achieving increasing effectiveness of embolization [5]. N-butyl cyanoacrylate (NBCA) is a liquid embolic agent that undergoes rapid polymerization upon contact with blood, resulting in immediate and permanent vascular occlusion [5]. Its flow-independent behavior allows deep penetration into the microvasculature, making it particularly effective in the management of hemorrhage, vascular malformations, and hypervascular tumors. Owing to these properties, NBCA has been increasingly applied in interventional radiology for the management of acute hemorrhage, vascular malformations, and hypervascular tumors, including selected cases of uterine artery embolization [6].

Therefore, we conducted a prospective single-arm study to evaluate uterine artery embolization (UAE) using N-butyl cyanoacrylate (NBCA) diluted with lipiodol at a ratio of 1:10 (1 part of NBCA to 10 parts of lipiodol) in patients with symptomatic uterine fibroids; hereafter referred to as the NBCA mixture. The objectives were to assess procedural and safety metrics, short-term clinical outcomes including symptom improvement and laboratory parameters, and fibroid volume changes on magnetic resonance imaging (MRI) after 3–6 months of follow-up.

## Methods

### Study design

This was a prospective, single-center, single-arm observational interventional study conducted at a tertiary referral university hospital to evaluate uterine artery embolization (UAE) using NBCA in patients with uterine fibroids requiring treatment. All patients were evaluated in a multidisciplinary setting involving gynecology and interventional radiology before treatment selection. The study protocol was approved by the institutional ethics committee, and written informed consent was obtained from all participants prior to enrollment. The study was conducted in accordance with the principles of the Declaration of Helsinki. From 2024 to 2025, forty consecutive women with uterine fibroids who met the predefined eligibility criteria were prospectively enrolled.

Inclusion criteria were: (1) age ≥ 18 years; (2) women with symptomatic uterine fibroids or asymptomatic enlarging fibroids considered appropriate for UAE following multidisciplinary evaluation by gynecologists and interventional radiologists; and (3) suitability for UAE based on clinical and imaging evaluation. Symptoms considered attributable to uterine fibroids included abnormal uterine bleeding, pelvic pain, bulk-related symptoms, or anemia secondary to fibroid-associated bleeding. Patients were included when they desired uterine preservation and/or were considered poor surgical candidates or declined surgical treatment after counseling regarding available therapeutic options.

Exclusion criteria were: (1) pregnancy or suspected pregnancy; (2) known or suspected gynecologic malignancy; (3) active pelvic infection; (4) severe allergy or contraindication to iodinated contrast media or Lipiodol; (5) severe comorbid conditions precluding endovascular intervention; (6) prior uterine artery embolization; (7) patients receiving ongoing fibroid-directed hormonal therapy.

No restriction was applied regarding fibroid size, number, or location. Pelvic MRI was performed before and after 3 to 6 months of intervention in all enrolled patients to confirm the diagnosis, characterize fibroid burden, and assess treatment suitability. MRI examinations were independently reviewed by two board-certified radiologists with experience in pelvic imaging. In case of discrepancy, a consensus interpretation was reached after joint review.

Patients with a desire for future fertility were specifically counseled regarding the uncertain reproductive outcomes after UAE and were managed according to institutional policy.

### Embolization technique

All procedures were performed by experienced interventional radiologists under local anesthesia and QL block (quadratus lumborum block). Post-procedural pain was managed using intravenous patient-controlled analgesia (PCA) according to the institutional protocol. Vascular access was obtained via the right radial artery using a 4-Fr introducer sheath, with a standardized transradial protocol including intra-arterial vasodilators and systemic heparinization.

A 4-Fr diagnostic catheter (MG2, Terumo, Japan) was used to perform sequential bilateral internal iliac angiography to assess uterine arterial anatomy and identify fibroid-feeding branches. The uterine arteries were then catheterized using the diagnostic catheter. Superselective catheterization of each uterine artery was achieved using a 1.9-Fr microcatheter (Tokai, Japan) advanced distally while avoiding non-target vessels.

Embolization was performed using N-butyl cyanoacrylate (NBCA) diluted with lipiodol at a ratio of 1:10. This dilution was selected to achieve a balance between adequate distal penetration, controlled polymerization time, and sufficient radiopacity, based on established principles of NBCA embolization balancing distal penetration and procedural control. Approximately 0.2 mL of the NBCA mixture was injected through the microcatheter, followed by a flush of 2 mL of 5% dextrose solution. This sequence was repeated 2–3 times until forward progression of the glue ceased and reflux was observed at the tip of the microcatheter, at which point the injection was stopped and the procedure advanced to the next step. Subsequently, NBCA was injected slowly under continuous fluoroscopic guidance. Limited reflux of the glue was permitted (approximately 2–3 cm proximal to the microcatheter tip) for up to 3 min, following the “plug-and-push” technique.

The angiographic endpoint was defined as cessation of forward glue progression within the tumor, with only reflux observed, indicating markedly reduced antegrade flow without further distal penetration. Bilateral uterine artery embolization was routinely performed unless contraindicated. However, in cases where unilateral embolization resulted in a spherical accumulation of embolic material within the fibroid, and contralateral angiography demonstrated no arterial supply to the tumor, embolization from a single uterine artery was considered sufficient. Technical success was defined as successful catheterization and embolization of all intended uterine arteries to the predefined endpoint, while preserving perfusion of the normal uterus. Hemostasis at the radial access site was achieved using a compression device.

### Outcome measures

Technical success was defined as successful embolization of all intended uterine arteries to the predefined angiographic endpoint. Safety outcomes included intraprocedural adverse events and post-procedural complications, classified according to the Society of Interventional Radiology (SIR) classification system. Post-embolization syndrome is characterized by a combination of the following pelvic pain, nausea, low-grade fever [7]. Post-embolization pain was assessed using a visual analog scale (VAS) within the first 24 h.

Secondary endpoints included procedural metrics, clinical outcomes (including symptom improvement and changes in hemoglobin levels in patients with baseline anemia), and imaging outcomes. Procedural metrics comprised total procedure time, fluoroscopy time, radiation dose (dose–area product), and length of hospital stay.

Clinical outcomes were assessed at 3–6 months after the procedure. Symptom improvement was evaluated based on patient-reported changes in specific fibroid-related symptoms, including abnormal uterine bleeding, pelvic pain, and bulk-related symptoms. Hemoglobin levels were reassessed at follow-up in patients with baseline anemia. The need for additional intervention, including repeat embolization or surgical treatment, was recorded.

Imaging outcomes were evaluated using pelvic MRI performed at 3–6 months after embolization. The dominant fibroid was selected as the target lesion for analysis. Fibroid volume was calculated using standardized methods, and percentage volume reduction was determined relative to baseline measurements.

### Statistical analysis

Continuous variables were expressed as mean ± SD or median (IQR), as appropriate. Categorical variables were presented as frequencies and percentages. Technical success, complications, and procedural metrics were analyzed descriptively. Changes in hemoglobin levels and MRI-derived fibroid volume between baseline and follow-up were analyzed using the paired t-test or Wilcoxon signed-rank test, as appropriate. Percentage fibroid volume reduction was calculated relative to baseline. A two-sided p value < 0.05 was considered statistically significant. No adjustment for multiple comparisons was applied because of the exploratory nature of the study. Statistical analyses were performed using IBM SPSS Statistics for Windows, version 24.0 (IBM Corp., Armonk, NY, USA).

## Results

### Patient characteristics

A total of 40 consecutive patients with uterine fibroids meeting the predefined eligibility criteria were prospectively enrolled. Baseline demographic, clinical, and imaging characteristics are summarized in Table 1. The study population primarily consisted of premenopausal women presenting with menorrhagia, pelvic pain, or bulk-related symptoms. Baseline anemia was documented in a subset of patients, for whom hemoglobin levels were systematically recorded prior to embolization.

**Table 1:** Baseline patient and fibroid characteristics

Variable	Value
Number of patients	40
Age (years), mean ± SD (range)	41 ± 6.5 (23–53)
Clinical presentation, n (%)	
Menorrhagia	31 (77.5)
Pelvic pain	5 (12.5)
Asymptomatic	7 (17.5)
Baseline hemoglobin (g/L), mean ± SD (range)	105.6 ± 22.2 (62–142)
Patients with baseline anemia, n (%)	24/40
Number of fibroids per patient	1.9 ± 1.5 (1–5)
Largest fibroid diameter (mm), mean ± SD (range)	64 ± 21.3 (16–118)
Dominant fibroid location n (%)	
Submucosal	11 (27.5)
Intramural	29 (72.5)
Subserosal	0 (0)

Clinical presentations were not mutually exclusive. Three patients presented with both menorrhagia and pelvic pain. Therefore, the total number of reported clinical presentations exceeds the number of patients

### Procedural metrics

Uterine artery embolization using NBCA was technically successful in all planned procedures. All procedures were completed through radial access without conversion to femoral access or significant access-site complications. Procedural parameters, including total procedure time, fluoroscopy time, radiation dose, embolic volume, and length of hospital stay, are detailed in Table 2. All procedures were completed via transarterial access with successful bilateral embolization when indicated.

**Table 2:** Procedural metrics

Parameter	Value
Technical success, n (%)	40 (100%)
Total procedure time (min), mean ± SD (range)	46.5 ± 13.3 (21–70)
Fluoroscopy time (min), mean ± SD (range)	17.1 ± 8.8 (7.9–43.3)
Radiation dose (DAP, μGy·m^2^), mean ± SD (range)	6161.0 ± 6425.5 (1784.9–23,916)
Length of hospital stay (days), mean ± SD (range)	1.9 ± 0.8 (1–3)

### Safety and periprocedural outcomes

No major intraprocedural adverse events were observed. Postprocedural complications are summarized in Table 3 and were predominantly minor in nature. Post-embolization pain was assessed using a visual analog scale (VAS) during the first 24 h after the procedure, with pain levels generally decreasing under standard analgesic management. Late adverse events included alopecia and vaginal dryness. No cases of non-target embolization or catheter entrapment were recorded.

**Table 3:** Periprocedural and postprocedural complications

Complication	n (%)
Intraprocedural adverse events, n (%)	0 (0%)
Early complications Fibroid expulsion, n (%)	1 (2.5)
Post-embolization syndrome, n (%)	8 (20)
Mean VAS pain score at 24 h, mean ± SD (range)	6.5 ± 1.4 (3–8)
Mean VAS pain score at 48 h, mean ± SD (range)	4.7 ± 1.3 (2–6
Fever, n (%)	0 (0%)
Transient vaginal bleeding, n (%)	0 (0%)
Late complications, n (%)	4 (10%)
Alopecia, n (%)	3 (7.5)
Vaginal dryness, n (%)	1 (2.5)

**Fig. 1:**
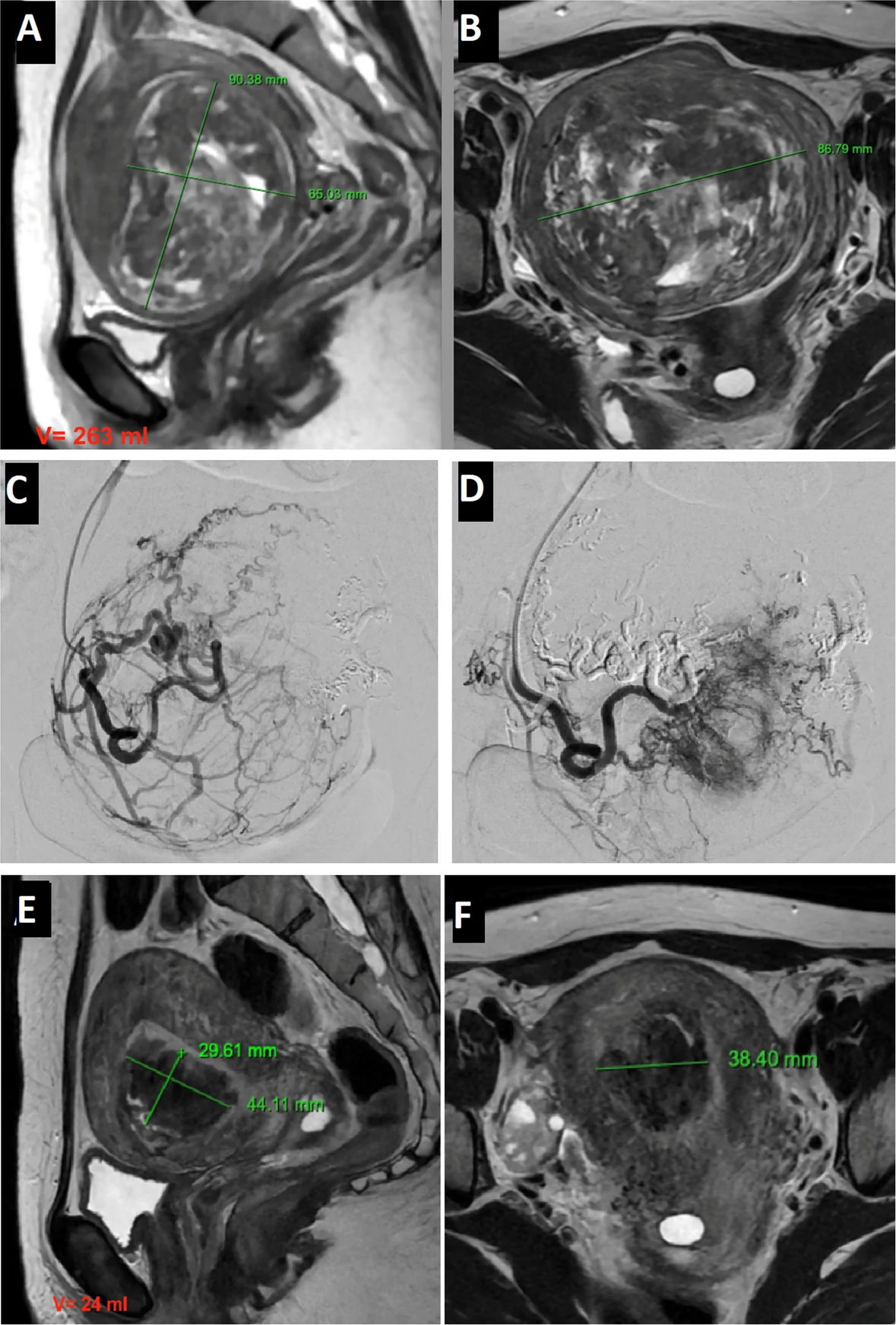
**A**, **B** Preprocedural T2-weighted MRI showing a large uterine leiomyoma. **C** Right uterine angiography demonstrating prominent arterial supply to the leiomyoma. **D** Post-embolization angiography following bilateral uterine artery embolization. **E**, **F** T2-weighted MRI obtained at the 3-month follow-up showing a marked reduction in fibroid volume from 263 to 24 mL, corresponding to a volume reduction of approximately 91%

The first illustrative case, there was a 40-year-old patient with a 89 × 64x85mm submucosal fibroid who underwent UAE (Fig. 1), after UAE 2 weeks the patient presented with abdominal pain, vaginal bleeding, tumor exteriorization vaginal. Examination suggested fibroid expulsion. Dilation and curettage were performed to remove the necrotic fibroid. There were no complications after removing the tumor. The patient was discharged on the same day. MRI obtained 3 months after embolization demonstrated a marked reduction in fibroid volume from 263 to 24 mL (Fig. 1).

**Fig. 2:**
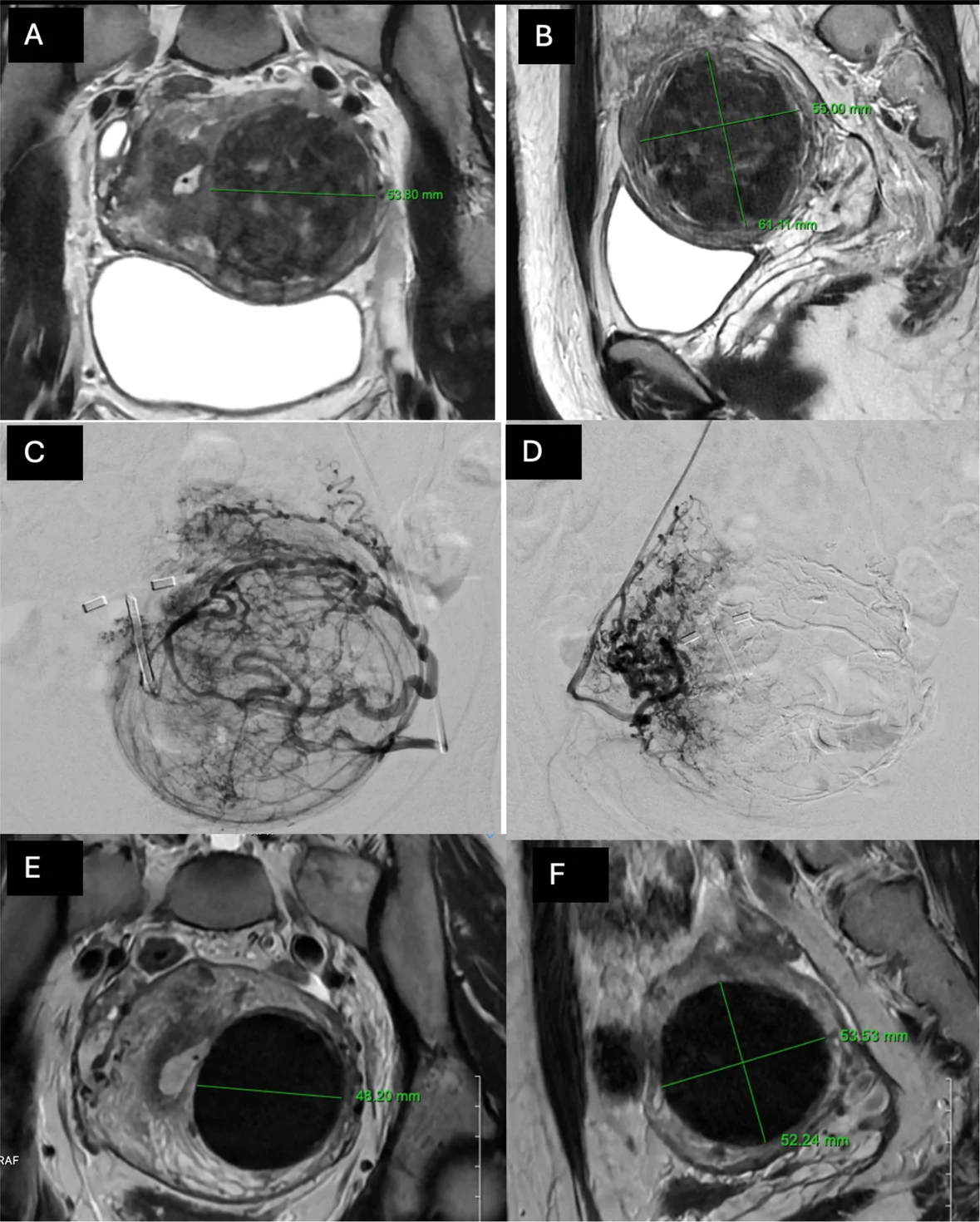
**A**, **B** Preprocedural T2-weighted MRI showing a large leiomyoma in the left uterine wall, measuring approximately 53.8 × 55.0 × 61.1 mm. **C** Left uterine angiography demonstrating prominent arterial supply to the fibroid. **D** Right uterine angiography showing minimal arterial supply to the fibroid; therefore, the right uterine artery was preserved. **E**, **F** T2-weighted MRI at 3-month follow-up showing a reduction in fibroid size to approximately 48.2 × 53.5 × 52.2 mm, with markedly decreased and more homogeneous T2 signal intensity, consistent with post-treatment changes

The second illustrative case involved a 35-year-old woman with a large leiomyoma located in the left uterine wall who desired future pregnancy. Digital subtraction angiography demonstrated that the fibroid was predominantly supplied by the left uterine artery. Selective embolization of the left uterine artery was therefore performed, while the right uterine artery was preserved because it provided minimal arterial supply to the fibroid. At the 3-month follow-up, T2-weighted MRI demonstrated a reduction in fibroid size from approximately 53.8 × 55.0 × 61.1 mm to 48.2 × 53.5 × 52.2 mm, together with markedly decreased and more homogeneous T2 signal intensity, consistent with post-treatment changes (Fig. 2).

## Clinical outcomes

Clinical follow-up was available for all patients at 3–6 months. Most patients reported improvement in fibroid-related symptoms, including reduction in menstrual bleeding and pelvic pain. Symptom improvement at 3–6 months observed in 100% patients and symptom disappearance at 3–6 months occurred in 87.5% patients. No patients required additional intervention.

### MRI and hemoglobin levels outcomes

All patients underwent pelvic MRI at 3–6 months after embolization. Quantitative MRI analysis demonstrated a significant reduction in fibroid volume compared with baseline. Fibroid volume measurements, hemoglobin levels and statistical comparisons are presented in Table 4. Paired analysis confirmed a significant decrease in fibroid volume at follow-up.

**Table 4:** MRI-based fibroid volume assessment

Parameter	Baseline	3–6 months	p value
Dominant fibroid volume (cm^3^), mean ± SD	137.7 ± 104.3 (11–490)	51 ± 54.3 (0–235)	< 0.001* (95% CI: 62.2–109.2)
Percentage volume reduction (%)		64.7 ± 22.7 (27–100)	
Hemoglobin change in anemic patients (g/L), mean ± SD (range)	92.6 ± 15.7	111 ± 9.3	< 0.001*

^*^Paired sample t-test

## Discussion

The prospective study provides a comprehensive evaluation of uterine artery embolization using N-butyl cyanoacrylate in patients with symptomatic uterine fibroids. The procedure, safety, clinical outcomes, and short-term magnetic resonance imaging (MRI) follow-up were assessed. Uterine artery embolization for the management of uterine fibroids was first reported by Ravina et al. [8] in 1995. Uterus preservation after UAE is achieved in nearly all women. Compared with the surgical group, the uterine artery embolization group had a shorter median hospital stay and a shorter time to return to work [9]. Approximately 20% of patients with UAE required an additional invasive procedure (hysterectomy or repeated uterine artery embolization) [9]. Some studies show that from 3 to 12 months after UAE, 3–100% fibroid volume reduction was observed [10, 11]. Besides technical success, the primary objective of the UAE is to improve symptoms. In the present study, most patients experienced improvement in fibroid-related symptoms, including menorrhagia and pelvic pain. The hemoglobin levels were improved. In the present study, most patients experienced improvement in fibroid-related symptoms, including menstrual bleeding abnormalities and pelvic pain.

Some types of materials are used: non-spherical, flaked polyvinyl alcohol (PVA), gelatin sponge (Gelfoam), and trisacryl gelatin microspheres (Embospheras), spherical particles of polyvinyl alcohol and polyvinyl acetate (PVA-PVAc) [12]. NBCA or glue has several advantages compared with other embolic materials. NBCA allows rapid permanent occlusion of vessels with lower recurrence and short procedure time. NBCA polymerizes almost instantly upon contact with blood, so effective embolization can often be achieved with fewer injections and repeated repositioning [13]. NBCA is commonly mixed with iodized oil (lipiodol). The advantage of NBCA is the NBCA polymerization time can be adjusted by changing the mixing ratio [13, 14]. More diluted NBCA travels further distally before polymerizing, leading more complete devascularization and less risk of recanalization or collateral circulation. For an experienced interventionist, this led to shorter overall procedure time. In this study, UAE using more diluted NBCA demonstrated efficient procedural performance. The ability of more diluted NBCA to penetrate distally into intratumoral vessels may contribute to faster embolization and reduced procedural complexity. This study provides the role of NBCA as an embolic agent in UAE. While embolic agents remain the most common materials, this study suggests that liquid embolics may represent a viable alternative in selected patients.

The selection of the NBCA–lipiodol dilution ratio is a critical determinant of embolization behavior, as it directly affects polymerization kinetics, viscosity, distal penetration, and procedural safety [15]. In the present study, a 1:10 ratio was chosen to achieve a balanced compromise between effective intratumoral penetration and controlled delivery. Increasing the proportion of lipiodol reduces the concentration of NBCA and modifies the physical properties of the mixture, allowing deeper distal migration into small intratumoral vessels; however, this may attenuate the embolic effect and increase the risk of unintended distal spread, including passage into non-target branches or pre-existing anastomoses such as ovarian arterial supply. Conversely, reducing the lipiodol proportion results in a more concentrated NBCA mixture with faster polymerization, which may lead to premature proximal occlusion, limited distal penetration, and an increased risk of microcatheter adhesion. Therefore, the choice of dilution must carefully balance these competing factors. In the broader embolization literature, more concentrated mixtures (e.g., 1:2–1:4) are typically used in high-flow conditions such as arteriovenous malformations to ensure rapid occlusion, whereas more diluted mixtures (e.g., 1:6–1:10 or higher) are preferred for tumor embolization to facilitate deeper penetration into the microvascular bed [15]. Although specific data on NBCA use in uterine artery embolization remain limited, these general principles are applicable, as the behavior of NBCA is governed more by its physicochemical properties and injection technique than by flow dynamics alone. In our experience, the 1:10 dilution provided a predictable and controllable injection profile under fluoroscopic guidance, enabling effective application of the “plug-and-push” technique while minimizing excessive reflux and non-target embolization. This suggests that a moderately more diluted NBCA mixture may be appropriate for achieving deep and homogeneous devascularization in uterine fibroids while maintaining procedural safety. The combination of diluted NBCA and transradial access may represent a simplified and efficient embolization strategy, particularly in cases requiring deep intratumoral penetration.

Major complications in a large meta-analysis were reported to be 2.9%; other complications included leiomyoma tissue passage (4.7%), deep venous thrombosis or pulmonary embolism (0.2%), and permanent amenorrhea (3.9%) [16]. The major complications are lower but the risk of ovarian failure is similar to surgery [17]. Minor complication rates up to 34% have been reported, usually related to the post-embolization syndrome (pyrexia, pain, and elevated inflammatory markers), vaginal discharge [9]. Major adverse events, is up to 15%, include severe vasovagal event requiring atropine; pelvic infection requiring readmission; fibroid expulsion; hematometria; persistent severe pain requiring hysterectomy — necrotic fibroid tissue seen on pathological analysis; pelvic abscess [9]. A study of Walker and Pelage [18] about 400 patients managed with UAE, 1% patients had infective complications requiring emergency hysterectomy, 7% patients had permanent amenorrhoea after embolisation, 4% women had chronic vaginal discharge considered as a major irritant. Endometrial atrophy occurs in 8.3% after UAE [12]. Fibroid expulsion has been reported after UAE, particularly in patients with submucosal or transmural leiomyomas [9, 19]. Although submucosal location may increase the risk of expulsion, it is not considered an absolute contraindication to UAE. Other late complications in our study include alopecia and vaginal dryness. Alopecia was reported by three patients during follow-up. Radiation-induced alopecia was considered unlikely because the radiation field was confined to the pelvis and did not involve the scalp. Although alopecia was temporally reported after UAE, a causal relationship with the procedure could not be established. Therefore, this finding should be interpreted with caution. Post-embolization pain is common following UAE. Although the mean VAS score at 24 h reflected moderate-to-severe pain intensity, pain was transient, adequately controlled with the standard analgesic regimen, and was not associated with prolonged hospitalization, readmission, additional invasive intervention, or permanent sequelae. Accordingly, it was classified as a minor adverse event based on its clinical consequences and the level of treatment required, rather than on pain intensity alone.

One strength of this study is its prospective design, as prospective data on the use of diluted NBCA–lipiodol mixtures for uterine artery embolization remain limited. However, several limitations should be acknowledged. First, this was a prospective single-arm study without a control group using conventional particulate embolic agents; therefore, comparative safety and efficacy cannot be established. Second, MRI follow-up was based on non-contrast imaging and focused on fibroid volume reduction rather than complete fibroid infarction. Previous studies have demonstrated that complete fibroid infarction on contrast-enhanced MRI is a stronger predictor of long-term clinical success than fibroid volume reduction alone, and that residual viable fibroid tissue may contribute to symptom recurrence [20]. Third, angiographic evaluation was limited to the uterine arterial circulation, and routine assessment of ovarian arterial supply, utero-ovarian anastomoses, or selective ovarian arteriography was not performed. Therefore, the potential contribution of collateral ovarian perfusion to treatment response or recurrence could not be evaluated. Finally, follow-up was limited to 3–6 months. Future prospective comparative studies incorporating contrast-enhanced MRI, comprehensive angiographic assessment of collateral blood supply, and longer clinical follow-up are warranted to further define the role of diluted NBCA–lipiodol mixtures in uterine artery embolization.

## Conclusion

This prospective single-arm study suggests that uterine artery embolization using a diluted NBCA–lipiodol mixture is feasible and appears to be safe, with favorable procedural characteristics and encouraging early clinical and MRI outcomes in patients with uterine fibroids. However, the short follow-up period, absence of a comparative control group, and lack of direct assessment of fibroid infarction limit the ability to draw definitive conclusions regarding its efficacy or its role relative to established particulate embolic agents. Further comparative studies with longer follow-up and systematic assessment of fibroid infarction are warranted.

## Data Availability

The datasets generated and/or analyzed during the current study are available from the corresponding author on reasonable request.
